# 152. Impact of BioFire FilmArray Blood Culture Identification Panel 2 (BCID2) and Antimicrobial Stewardship Interventions on Time to Optimal Antimicrobial Therapy in Patients with Positive Blood Cultures

**DOI:** 10.1093/ofid/ofad500.225

**Published:** 2023-11-27

**Authors:** Isaac Nichols, Anjly Kunapuli

**Affiliations:** Oklahoma State University Medical Center, Pryor Creek, Oklahoma; Oklahoma State University Medical Center, Pryor Creek, Oklahoma

## Abstract

**Background:**

Delayed treatment of bloodstream infections is associated with increased morbidity and mortality. Conventional methods for organism identification and susceptibility data from blood cultures can take about 2-5 days. Technological advancements in gene-based polymerase chain reaction tests amplify DNA targets from positive blood cultures and can shorten the identification time of certain organisms and resistance genes, aiding in earlier optimization of antimicrobial therapy.

**Methods:**

This pre/post quasi-experimental, single center study was conducted 6 months prior to and 6 months after implementation of the BCID2 panel. Patients with at least 1 positive blood culture with an organism on the BCID2 panel were included in the study. One hundred twenty-five patients were included in the pre-BCID2 arm and 114 patients in the BCID2 arm. In the BCID2 panel group, real-time alerts were received by the antimicrobial stewardship team (AST) and recommendations were made to providers. The primary outcomes were time to pathogen identification, effective therapy, and optimal therapy.

**Results:**

Demographic characteristics were similar in both groups. A higher percentage of patients with multiple risk factors for bacteremia were identified in the pre-BCID2 arm (36.8% vs 20.2%).

The mean time to pathogen identification (60 hrs vs 30 hrs; *P* < 0.0001) and time to optimal therapy (61.4 hrs vs 44.6 hrs; *P*=0.001) were shorter in the BCID2 arm compared to the pre-BCID2 arm. Patients in the BCID2 arm had an overall trend toward decreased mean time to effective therapy compared with the pre-BCID2 arm (17.5 hours vs. 14 hours; *P*=0.18). Subgroup analysis of blood cultures with vancomycin-resistant *Enterococcus spp*., ESBL-producing organisms, and *Candida glabrata* met statistical significance in all three primary outcomes. The BCID2 arm had a 21.9 hr. reduction in time to pathogen ID (49.6 v. 27.7; *P*=0.02), 38.9 hr. reduction in time to effective therapy (66.9 vs. 28; *P*=0.013), and a 54.5 hr. reduction in time to optimal therapy (89.3 vs 34.8; *P*=0.046). Overall, 99% of recommendations from the AST were accepted by providers.

Primary Outcome Results

**Figure:**
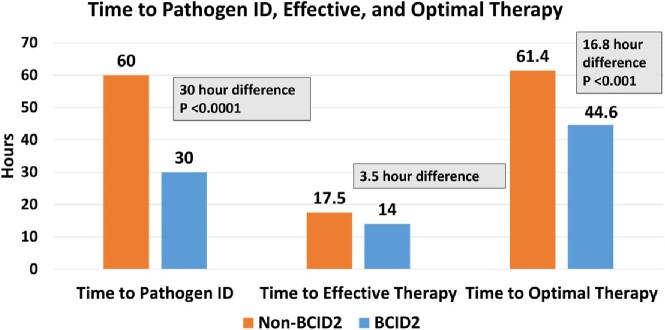

Time to pathogen ID, Time to Effective Therapy, Time to Optimal Therapy

Secondary Outcomes 1

**Figure:**
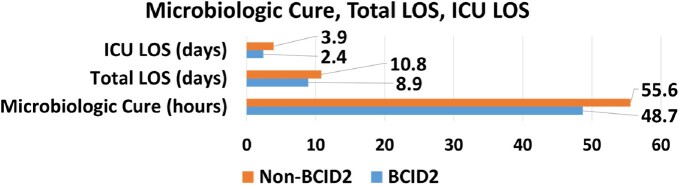

Total length of stay, ICU length of stay, Microbiologic Cure

Secondary Outcomes 2

**Figure:**
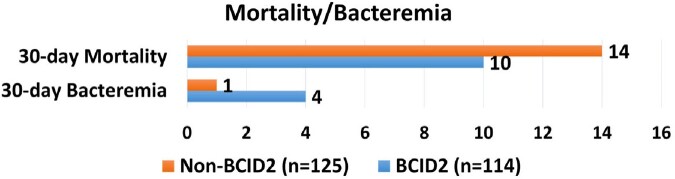

30 day Mortality and Recurrent bacteremia

**Conclusion:**

Implementation of the BCID2 panel significantly decreased time to pathogen identification and optimal therapy in patients with bacteremia.

**Disclosures:**

**All Authors**: No reported disclosures

